# PLC-Based Polymer/Silica Hybrid Inverted Ridge LP_11_ Mode Rotator

**DOI:** 10.3390/mi15060792

**Published:** 2024-06-16

**Authors:** Jiaqi Liang, Daming Zhang, Xinyu Lv, Guoyan Zeng, Pai Cheng, Yuexin Yin, Xiaoqiang Sun, Fei Wang

**Affiliations:** State Key Laboratory of Integrated Optoelectronics, College of Electronic Science and Engineering, Jilin University, Changchun 130012, China; liangjq21@mails.jlu.edu.cn (J.L.); zhangdm@jlu.edu.cn (D.Z.); lvxy21@mails.jlu.edu.cn (X.L.); zenggy21@mails.jlu.edu.cn (G.Z.); chengpai22@mails.jlu.edu.cn (P.C.); yxyin@jlu.edu.cn (Y.Y.); sunxq@jlu.edu.cn (X.S.)

**Keywords:** mode division multiplexing, mode rotator, optical communication, polymer optical waveguide

## Abstract

The mode rotator is an important component in a PLC-based mode-division multiplexing (MDM) system, which is used to implement high-order modes with vertical intensity peaks, such as LP_11b_ mode conversions from LP_11a_ in PLC chips. In this paper, an LP_11_ mode rotator based on a polymer/silica hybrid inverted ridge waveguide is demonstrated. The proposed mode rotator is composed of an asymmetrical waveguide with a trench. According to the simulation results, the broadband conversion efficiency between the LP_11a_ and LP_11b_ modes is greater than 98.5%, covering the C-band after optimization. The highest mode conversion efficiency (MCE) is 99.2% at 1550 nm. The large fabrication tolerance of the proposed rotator enables its wide application in on-chip MDM systems.

## 1. Introduction

Increasing internet traffic drives the transmission capacity of optical communication networks dramatically. The capacity of the communication system using a single-mode fiber (SMF) has reached its limit [1,2]. Therefore, mode-division multiplexing (MDM) technology, a kind of space-division multiplexed (SDM), is a promising technology that can increase the transmission capacity by transmitting separate signals in a few-mode fiber (FMF) with orthogonal optical modes [3,4]. Integrated optics, compatible with large-volume and low-cost complementary metal oxide semiconductor (CMOS) technology, is a method to realize compact and complex on-chip systems. Currently, MDM components, including mode-division multiplexer/demultiplexer (MUX/DeMUX) [5,6,7,8], mode-selective switch [9,10,11], and multimode spirals [12,13], have been proposed and applied in reconfigurable optical networks [14,15], optical computation [16], and nonlinear optics. However, traditional waveguides are co-planar (or 2D) structures that impose significant limitations on the design of (de-)multiplexers with a three-dimensional (3D) spatial distribution of fiber modes [17]. For example, LP_11b_ mode light cannot be excited by co-planar structures of the same thickness. Here, we define the LP_11a_ mode as the mode with two intensity peaks in the plane and the LP_11b_ mode as the mode with two intensity peaks in the vertical direction. To address this issue, various methods have been proposed and experimentally validated, such as mode rotators [18,19,20,21,22,23,24], vertical asymmetric directional couplers [25,26,27], horizontal directional couplers formed with waveguides of different heights [28], and degenerate-mode-selective couplers [29]. Polymer-based planar light-wave circuits (PLCs) offer a simple, low-cost, and flexible fabrication process [30,31,32] compared to silica and silicon. Additionally, the polymer/silica hybrid waveguide offers a platform combining the advantages of both materials to achieve stable and low-power-consumption optical devices [33,34]. Filling a polymer into a silica trench to form waveguides is an efficient method to reduce the loss introduced by sidewall roughness and rounded corner effects due to annealing [35,36,37]. The mature fabrication of silica etching makes polymer/silica hybrid inverted ridge waveguide suitable for on-chip MDM systems. Furthermore, a polymer with high thermos-optic coefficients (TOC) has the potential to reconfigure MDM systems.

In this paper, we designed a polymer/silica hybrid inverted ridge waveguide mode converter to realize the conversion between the LP_11a_ and LP_11b_ modes. Numerical simulations show that the conversion between LP_11a_ (LP_11b_) and LP_11b_ (LP_11a_) modes achieves a conversion efficiency greater than 98% throughout the C-band, with a maximum mode conversion efficiency of 99.2% at 1550 nm. The mode rotator also shows a good fabrication tolerance.

## 2. Principle and Design

**Figure 1:**
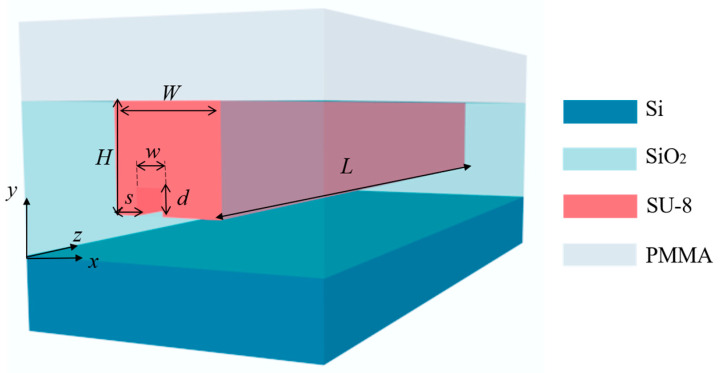
Three-dimensional (3D) schematic of the mode rotator.

Figure 1 depicts the 3D schematic of the proposed mode rotator, which consists of a SiO_2_ buffer layer, an SU-8 core, and a polymethyl-methacrylate (PMMA) cladding. The refractive indices of silica, SU-8, and PMMA are 1.4448, 1.5802 and 1.4456, respectively. The large refractive index difference between SU-8 and silica causes spot mismatch when light is transmitted from the fiber to the waveguide, but this can be solved by introducing SSC [38] or using a high-NA fiber for coupling. This paper focuses on the design and tolerance of the rotators. This mode rotator structure is a multimode rectangular straight waveguide with a straight trench of width *w*, depth *d*, and position *s*. The optical mode is not disturbed without the introduction of the silica trench.

**Figure 2:**
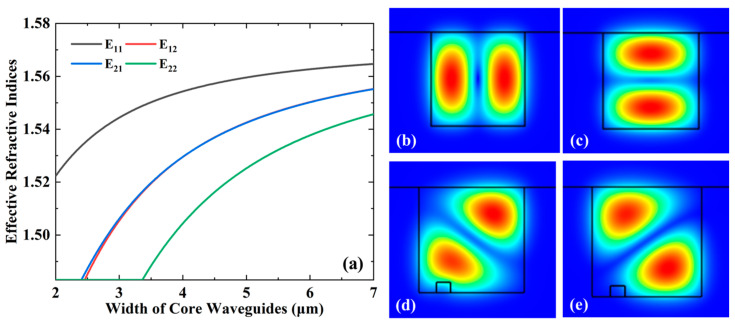
(**a**) Relationships between effective refractive indices of different modes with widths of core waveguides at 1550 nm while keeping *H* = *W*; optical mode distributions for the mode rotator (**b**) LP_11a_(E_21_) mode; (**c**) LP_11b_(E_12_) mode; (**d**) 1st LP_11_ mode; (**e**) 2nd LP_11_ mode.

We analyze the relationships between the effective refractive indices of different modes with widths of core waveguides at 1550 nm, while *W* is equal to *H*, as shown in Figure 2a. The number of transmitted modes in a waveguide is positively related to the size of the waveguide. Based on our previous experimental results [33,34], a core layer geometry of 3 × 3 μm^2^ is suitable for fundamental mode propagation. To minimize coupling losses with subsequently connected mode (de)multiplexer devices while realizing the multimode conditions, we set the waveguide width *W* = 6 μm and height *H* = 6 μm. There are many modes that can be propagated in a large core geometry, which increases the loss caused by the modes. However, high-order modes should be exited with special designs, such as asymmetric directional coupler-based mode multiplexers. The modes inside the rotator can be controlled precisely. The optical mode field distributions of LP_11a_ and LP_11b_ are shown in Figure 2b,c. The angle of transmission of light in the waveguide can be controlled by changing the parameters and positions of the trench, which will further control the conversion degree of the optical modes. To achieve a 90-degree rotation from LP_11a_ to LP_11b_, two orthogonal LP_11_ modes with the optical axes rotated by 45° with respect to the x- and y-axes need to be equivalently excited in the straight waveguide, as shown in Figure 2d,e. The mode fields are different between devices with and without trenches, contributing to larger mode mismatches and higher crosstalk at the boundary as the trench size increases. There is a clear trade-off between the parameter of the trench and the rotator performance. However, as the depth and width of the trench increase, the loss and crosstalk increase. Therefore, in our design, we choose the target parameters of *w* = 0.8 μm and *d* = 0.6 μm to realize the lower loss and high mode conversion efficiency (MCE). The propagation constants in the waveguide for the two orthogonal LP_11_ modes are, respectively, *β*_1_ and *β*_2_. By setting the length *L* of the waveguide with the trench to a half-beat length, *L = π*/(*β*_1_ − *β*_2_), the LP_11a_ (LP_11b_) mode is rotated into the LP_11b_ (LP_11a_) mode.

**Figure 3:**
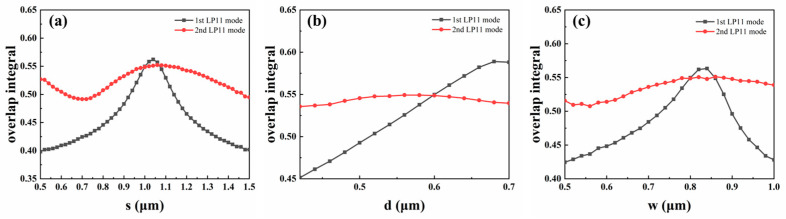
(**a**) Trench position *s*; (**b**) trench depth *d*; and (**c**) trench width *w* dependence of normalized overlap integral of 1st and 2nd LP_11_ modes shown in Figure 2c,d, with LP_11a_ mode at a wavelength of 1550 nm.

Next, we optimize the trench parameters using the normalized overlap integration method based on the assumed values. The 1st and 2nd LP_11_ modes are excited, while the normalized overlap integrals between the LP_11a_ with the 1st LP_11_ modes and the LP_11a_ with the 2nd LP_11_ modes are the same. Therefore, we summarize the relationships between the normalized overlap integrals and trench parameters at 1550 nm, as illustrated in Figure 3a. Figure 3a shows the relationship between *s* and the normalized overlap integrals, while *w* = 0.8 μm and *d* = 0.6 μm. As shown in Figure 3a, two values of *s* are taken to make the LP_11a_ mode overlap and be equivalent to the two orthogonal LP_11_ modes. To reduce the size of the trench, *s* is chosen to be 1 μm. Figure 3b,c show *d* and *w* dependence of the normalized overlap integral of the LP_11a_ mode with 1st and 2nd LP_11_ modes at a wavelength of 1550 nm, respectively. In Figure 3b, *w* = 0.8 μm and *s* = 1 μm, and in Figure 3c, *s* = 1 μm and *d* = 0.6 μm. From the calculation results, it can be seen that the variation in the trench parameters changes the normalized overlap integral of the LP_11a_ mode with the 1st and 2nd LP_11_ modes. Finally, the length of the mode rotator *L* is calculated to be *L* = 977 μm based on the half-tap length equation described above, according to the parameters *w* = 0.8 μm, *d* = 0.6 μm, and *s* = 1 μm.

## 3. Optimization Results and Character

**Figure 4:**
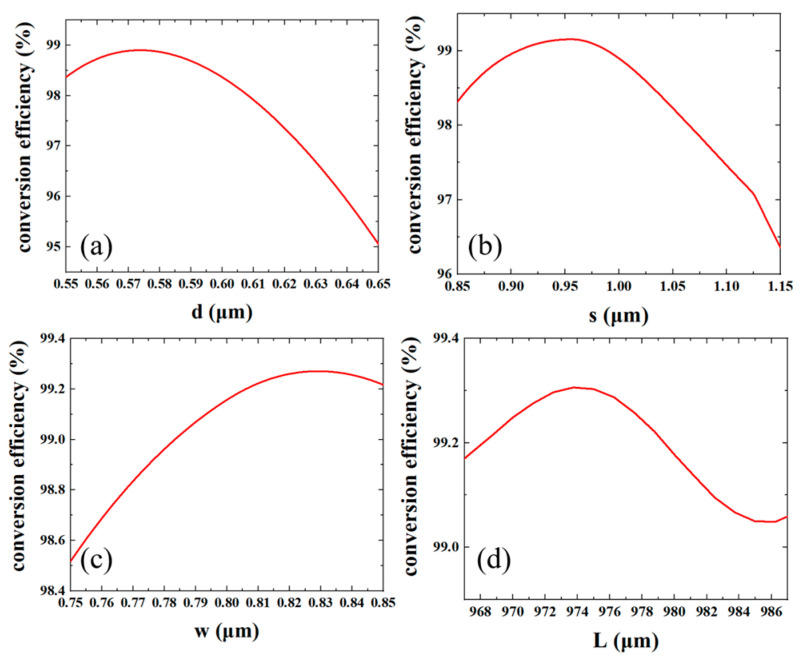
Optimization of (**a**) trench depth *d*; (**b**) trench position *s*; (**c**) trench width *w*; (**d**) mode rotator length *L*.

In order to improve the MCE, the parameters are further optimized. First, the width and position of the trench are fixed, and the depth *d* is optimized. As shown in Figure 4a, the conversion efficiency is optimized when *d* is 0.576 μm. Then, as shown in Figure 4b, we scanned the position s of the trench from the edge and obtained an optimal distance *s* of 0.955 μm. After obtaining the optimal *d* and *s*, the width of trench *w* is optimized as shown in Figure 4c, and the optimal value of *w* is 0.83 μm. Finally, the length of the mode rotator, *L*, is optimized after the parameters of the trench have been determined. As shown in Figure 4d, the best results are obtained when the length of the mode rotator is 973 μm. The optimized parameters of the mode rotator are summarized in Table 1.

**Figure 5:**
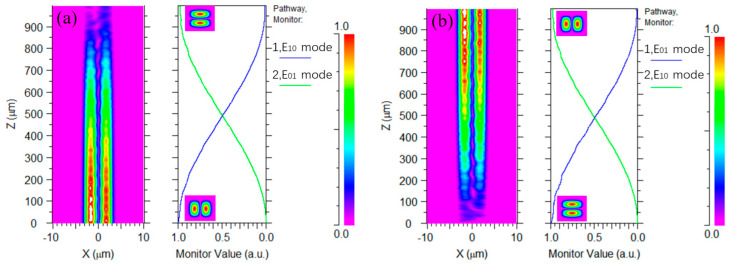
(**a**) Simulated modal transmission based on optimized parameters of the LP_11a_ mode when launched at 1550 nm; (**b**) simulated modal transmission based on optimized parameters of the LP_11b_ mode when launched at 1550 nm.

We used the beam propagation method (BPM) using the Rsoft software (Rsoft 8.0). As shown in Figure 5, the MCE from the LP_11a_ (LP_11b_) mode to the LP_11b_ (LP_11a_) mode at 1550 nm is improved to 99.2% by simulating and optimizing the size of the mode rotator. From the light-field transmission in Figure 5, the LP_11a_ mode component is clearly visible, but not the LP_11b_ mode component. This is because the field is zero at the center of the core in the vertical direction.

**Figure 6:**
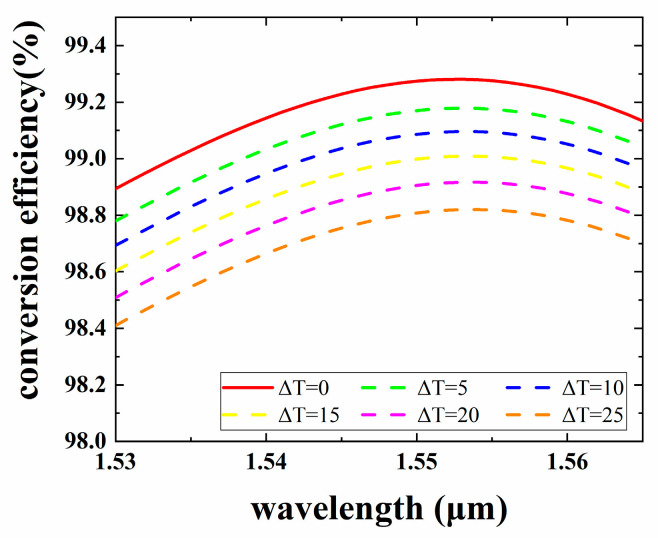
Conversion efficiency of the mode rotator proposed over the C-band differed with the launch of the LP_11a_ mode.

Because the polymer has a relatively larger TOC, the effect of temperature changes on the MCE of the device needs to be considered. Figure 6 shows the wavelength dependence of the rotator in the C-band (1530 nm to 1565 nm) at different temperatures. Since the dispersion of the materials is very small, the variation can be neglected at short wavelengths. As shown in Figure 6, this rotator is capable of mode conversion in the C-band and has an MCE greater than 98.5%. Thus, the wavelength dependence of the MCE in the C-band is negligible. The simulation data were obtained at a room temperature of 298.15 K. The MCE decreased by 0.4% when the temperature was increased by 25 K.

**Figure 7:**
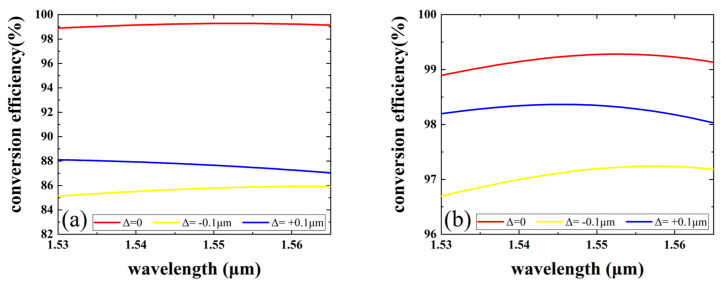
Fabrication tolerance to (**a**) trench depth *d* and (**b**) trench width *w*.

In this study, the polymer materials, SU-8 and PMMA, are chosen as the core and cladding films for the mode rotator. The processes for the mode rotator, including wet etching and inductively coupled plasma (ICP) etching, introduce unexcepted fabrication variations in trench width and position. At the same time, the nonlinear variation in the etching rate affects the depth of the trench. Therefore, fabrication errors are introduced. Manufacturing errors change the magnitude of the normalized overlap integral of the LP_11a_ mode with the 1st and 2nd LP_11_ modes, which changes the angle of rotation of the optical field and ultimately affects the device conversion efficiency. Figure 7 shows the manufacturing error of the mode rotator when the LP^11a^ mode is emitted at 1550 nm. We first discuss the effect of trench depth variation on the conversion efficiency. Figure 7a depicts the numerical results of the effect of the variation in the trench depth d on the MCE. The figure shows that ∆*d* is set to 0 and ±0.1 µm, and when d is set to ±0.1 µm, the MCE is substantially reduced to 85%. As shown in Figure 7b, the MCE of the mode rotator is greater than 96.6% when the trench width w is varied within ±0.1 µm design parameters. So, the width of this device can still be considered to have large process tolerances.

## 4. Discussion

Table 2 shows a comparison of the mode rotators demonstrated in recent years. The introduced trench is a normal method for mode rotation. In [18], a silica-based rotator is experimentally demonstrated to benefit from mature fabrication techniques. To further optimize device performance, optimization algorithms are used in [19]. However, the introduction of a trench increases the complexity of fabrication. In [20], the rotator is achieved by a heater. Thermally induced asymmetric refractive index distribution via the TO effect in the horizontal and vertical directions makes the mode rotation between modes possible when the heater is “ON” but impossible when the heater is “OFF”. The power consumption is high at 161.5 mW, although the core is a high thermal optical coefficient (TOC) polymer. The thermal crosstalk influences the other components in the same chip. The proposed rotator with multiple tapered trenches [21] has many advantages in terms of MCE and process tolerance. However, due to the continuous change in the cross-section, the fabrication process is more complicated compared to a rotator with a simple L-shaped waveguide. Our proposed device shows a compact footprint and broadband MCE by optimizing a straight trench. The simulation results after parameter optimization show that the conversion between the LP_11a_ and LP_11b_ modes can be greater than 98.5% over the C-band, with a maximum MCE of 99.2% at 1550 nm.

## 5. Conclusions

In conclusion, we have designed an LP_11_ mode rotator based on a polymer/silica hybrid inverted ridge waveguide, which enables conversion between the LP_11a_ and LP_11b_ modes. The simulation results, after parameter optimization, show that the conversion between LP_11a_ and LP_11b_ modes can be achieved with an MCE exceeding 98.5% over the C-band, with a maximum MCE of 99.2% at 1550 nm. Additionally, we have also verified that this model rotator has a high fabrication tolerance. The designed mode rotator can be connected to other devices embedded in the waveguide structure to achieve multimode multiplexing and increase the integration of the device.

## Figures and Tables

**Table 1:** Optimized design parameters of the mode rotator.

*W*	*H*	*s*	*d*	*w*	*L*
6 μm	6 μm	0.955 μm	0.576 μm	0.83 μm	973 μm

**Table 2:** Performance comparison of mode rotator.

Structure	Wavelength Range (nm)	Materials	MCE (%)	Structure	Volumetric (μm^3^)	Electric Power (mW)
[18]	1450~1560	silica Δ = 0.45%	>90%	straight trench	11.3 × 11 × 1460	N.A.
[19]	1550	silica Δ = 1%	98.7%	curved trench	10 × 10.1 × 1000	N.A.
[20]	1530~1610	polymer	>84%	heater	8 × 8 × 650	161.5
[21]	1500~1600	silica Δ = 1%	99.4%	tapered trenches	10 × 8.6 × 2000	N.A.
This work	1530~1565	Polymer Δ = 8.5%	>98.5%	straight trench	6 × 6 × 973	N.A.

N.A., not applicable.

## Data Availability

Data are contained within the article.
